# Herpes Zoster and Post-Herpetic Neuralgia—Diagnosis, Treatment, and Vaccination Strategies

**DOI:** 10.3390/pathogens13070596

**Published:** 2024-07-17

**Authors:** Delwyn Zhi Jie Lim, Hong Liang Tey, Brenda Mae Alferez Salada, Jolene Ee Ling Oon, Ee-Jin Darren Seah, Nisha Suyien Chandran, Jiun Yit Pan

**Affiliations:** 1 National Skin Centre, Singapore 308205, Singapore; teyhongliang@ntu.edu.sg (H.L.T.); jypan@nsc.com.sg (J.Y.P.); 2 Lee Kong Chian School of Medicine, Nanyang Technological University, Singapore 637371, Singapore; 3 Yong Loo Lin School of Medicine, National University of Singapore, Singapore 169857, Singapore; jolene_oon@nuhs.edu.sg (J.E.L.O.); nisha_suyien_chandran@nuhs.edu.sg (N.S.C.); 4 Division of Infectious Diseases, University Medicine Cluster, National University Health System, Singapore 119228, Singapore; brenda_mae_alferez@nuhs.edu.sg; 5 National Healthcare Group Polyclinics, Singapore 308433, Singapore; darren_ej_seah@nhgp.com.sg; 6 Division of Dermatology, Department of Medicine, National University Hospital, Singapore 119074, Singapore

**Keywords:** herpes zoster, post-herpetic neuralgia

## Abstract

Introduction: Herpes zoster is caused by the reactivation of latent varicella infection within the sensory ganglia, caused by the varicella-zoster virus (VZV). The disease is classically characterized by a painful unilateral vesicular eruption. Complications of the disease include herpes zoster ophthalmicus, Ramsay Hunt syndrome, acute retinal necrosis, and post-herpetic neuralgia. In this paper, we discuss the epidemiology, pathogenesis, clinical features, diagnosis, management, and vaccination strategies of herpes zoster and post-herpetic neuralgia. Method: This paper was developed with input from specialists from Singapore’s public sectors—dermatologists, family physicians, and infectious diseases specialists. Results: The diagnosis of herpes zoster is clinical and can be aided with laboratory investigations. Early initiation of antivirals, within 72 h of onset, can reduce the severity and duration of the condition and decrease the intensity of pain. In patients with a high risk of post-herpetic neuralgia, early initiation of anticonvulsants or tricyclic antidepressants can be considered. Herpes zoster is highly preventable, with the advent of the recombinant zoster vaccine (RZV) providing an overall vaccine efficacy of 97.2%. Procedures such as epidural blocks and subcutaneous or intracutaneous injections of local anesthetics and steroids can be considered for patients with a high risk of post-herpetic neuralgia to reduce its incidence. Conclusion: This article serves as a guideline for clinicians in the diagnosis, investigations, management, and prevention of herpes zoster. With the majority of adults in Singapore currently at risk of developing herpes zoster due to varicella immunization being only introduced in 2020, it is important for clinicians to recognize and manage herpes zoster appropriately.

## 1. Introduction

Herpes zoster, or shingles, is caused by the varicella-zoster virus (VZV), also known as human herpes virus 3 (HHV 3). It represents the reactivation of latent varicella infection within the sensory ganglia. Cutaneous features of the disease are characterized by a unilateral painful vesicular eruption over a restricted dermatome [1]. Early initiation of antiviral treatment reduces complications of the disease. Herpes zoster-associated pain tends to resolve over time, but some patients suffer from post-herpetic neuralgia (PHN). PHN is characterized by neuropathic pain lasting beyond the resolution of the rashes. In this paper, we discuss the epidemiology, pathogenesis, clinical features, diagnosis, management of zoster and PHN, and vaccination strategies against herpes zoster.

## 2. Methods

This guideline was developed with the input from specialists from Singapore’s public sectors who are involved in the diagnosis and management of herpes zoster and PHN, namely dermatologists, family physicians, and infectious diseases specialists.

The group of authors realized a need for such a guideline as the incidence of herpes zoster and post-herpetic neuralgia will increase worldwide with an aging population. The scope of the article was then agreed upon. A literature review of all articles and guidelines related to herpes zoster and post-herpetic neuralgia was performed and the most relevant articles were selected for review.

## 3. Results

### 3.1. Epidemiology

Herpes zoster is classically a disease of adulthood, affecting those older than 50 years old, although it can occur in younger persons and in particular those who had primary varicella infection within the first year of life [2]. Individuals with a history of primary varicella have a 30% lifetime risk of developing zoster [3]. The severity and incidence of herpes zoster increase significantly with age, in view of a specific age-related decline in cell-mediated immunity to the varicella-zoster virus. Risk factors for the development of herpes zoster include advancing age, immunosuppression states, concomitant chronic diseases, and the presence of physical trauma (Table 1) [4,5,6].

In Singapore, varicella immunization was only included in the national childhood immunization schedule in 2020. Prior studies have shown that the seroprevalence of varicella zoster amongst Singapore adults over the age of 25 is high at 88% from 1998 to 2010 [7]. This would suggest that the majority of adults currently are at risk of developing herpes zoster.

Risk factors for post-herpetic neuralgia are listed in Table 2. The risk of PHN is not increased in immunocompromised individuals.

### 3.2. Pathogenesis

#### 3.2.1. Herpes Zoster

Acute herpes zoster is caused by the reactivation of VZV. During initial infection, a cell-free virus, which is present only in skin vesicles, infects nerve endings in the skin and migrates along sensory axons to establish latency in the neuron within the regional ganglion [8]. After the resolution of the original infection, the virus persists in the dorsal root ganglia of cranial or spinal nerves. When cellular immunity diminishes, VZV reactivation occurs and the virus is transported along the peripheral nerves, producing an acute neuritis. Concurrently, the dorsal ganglion exhibits intense inflammation with hemorrhagic necrosis of nerve cells. The ganglion undergoes eventual neuronal loss with fibrosis of afferent nerve fibers, especially type C nociceptors. The dermatomal distribution of herpes zoster corresponds to the sensory fields of many infected afferent neurons and their associated specific ganglion.

#### 3.2.2. Post-Herpetic Neuralgia

There are a few mechanisms in which PHN develops [9,10]:

1. Upon damage of the nerves, roots, and ganglion, these peripheral nerves lose the ability to inhibit nociceptive pain signals. This lowers the threshold for nociceptive pain activation and produces spontaneous ectopic discharges, generating disproportionate pain with non-painful stimuli;
2. With small fiber deafferentation from damage, the C fibers become sensitized and lower their threshold for action potentials. This increases their discharge rate and magnitude, resulting in peripheral nervous system-mediated spontaneous pain and allodynia;
3. Lastly, there are some patients who experience constant pain in a region of profound sensory loss without allodynia, also termed anesthesia dolorosa. In these patients, there is loss of both large and small diameter fibers and the pain is likely due to intrinsic central changes with increased spontaneous activity in deafferented central neurons and/or reorganization of central connections.

### 3.3. Clinical Features

#### 3.3.1. Rash

Herpes zoster patients usually present acutely with a painful vesicular rash in a dermatomal distribution. The pain can precede the appearance of the rash by 2–3 days [11], making it difficult to diagnose during the prodromal stage. The rash presents initially as erythematous macules or papules, which progresses to vesicles and pustules over three to four days. In immunocompetent hosts, the lesions usually crust in seven to ten days. In immunocompromised patients, the rash may become disseminated and/or associated with visceral organ involvement (e.g., pneumonia or encephalitis) [12,13,14].

Zoster rash may also present in the following two ways:

1. Multi-dermatomal zoster: less than 20 lesions involving adjacent 2 or 3 dermatomes;
2. Disseminated zoster: more than 20 vesicles outside the area of the primary and adjacent dermatomes or involving other systemic organs [15].

#### 3.3.2. Complications

Acute herpes zoster occurring at certain sites may also predispose the patient to certain complications. These complications are listed in Table 3.

### 3.4. Diagnosis and Investigations

Herpes zoster is a clinical diagnosis, especially once the typical cutaneous features are present. However, in immunocompromised patients, the presentation may be atypical. An important differential diagnosis to consider is herpes simplex virus infection [11].

When laboratory investigation is indicated, polymerase chain reaction (PCR) testing is preferred (>95% sensitive, 99% specific) and can be used for both cutaneous and non-cutaneous manifestations [12,13]. Vesicles or erosions seen in cutaneous infections should be swabbed and sent for PCR testing. In non-cutaneous infections, PCR should be performed on the fluids of the organ involvement (e.g., cerebrospinal fluid in meningitis or vitreous samples in acute retinal necrosis).

### 3.5. Treatment

#### 3.5.1. Herpes Zoster

In general, the treatment goals of herpes zoster are reducing the progression of cutaneous lesions, decreasing the intensity of acute zoster pain, and lessening the incidence of postherpetic neuralgia. Treatment of immunocompromised hosts is also intended to reduce the risk of associated complications.

Treatment of acute herpes zoster is divided into whether it is an uncomplicated or complicated zoster. An uncomplicated zoster is defined as a zoster affecting two dermatomes in immunocompetent hosts at most. A complicated zoster is defined as a zoster occurring in immunocompromised hosts, a disseminated zoster (regardless of immune status), or a zoster occurring at special sites (e.g., herpes zoster ophthalmicus/opticus or with neurological complications).

An uncomplicated zoster can be treated with oral antivirals (e.g., acyclovir and valacyclovir) and is recommended to be initiated within 72 h of clinical symptoms [11]. Antiviral therapy can still be considered after 72 h if there are still new lesions at the time of presentation or if there are lesions at a site associated with an increased risk of complications, e.g., ocular involvement [16]. In complicated zoster and all immunocompromised hosts, treatment can still be administered after 72 h with intravenous acyclovir as the initial agent (10–15 mg/kg body weight every 8 h) for 10 to 14 days duration [11].

For zoster at special sites:

- In herpes zoster ophthalmicus, antiviral treatment is always recommended, even beyond 72 h of onset. Patients should also be referred to an ophthalmologist to exclude ocular involvement, especially if there is a positive Hutchinson sign (zoster lesions on the tip, sides, and root of the nose). The standard approach to herpes zoster ophthalmicus includes oral antiviral therapy. However, intravenous acyclovir should be administered if the patient is immunocompromised or requires hospitalization for sight-threatening disease;
- In acute retinal necrosis, treatment includes intravenous acyclovir for 10 to 14 days, followed by oral valacyclovir 1 g three times daily (or equivalent) for approximately six weeks. In addition, systemic glucocorticoids may be required;
- In Ramsay Hunt syndrome, treatment includes oral valacyclovir and oral prednisolone (1 mg/kg for five days, without a taper). In severe cases such as the presence of vertigo, tinnitus, or hearing loss, intravenous acyclovir can be initiated and transmitted to an oral antiviral agent when lesions crust.

The usual dosing of antiviral medications can be found in Table 4.

#### 3.5.2. Pain Control in Acute Herpes Zoster

Adequate pain control is important in herpes zoster. A combination of antiviral therapy with effective relief of acute pain may lessen the risk of post-herpetic neuralgia (PHN) since severe acute pain is a risk factor for PHN [17].

To our best knowledge, there are no good published data on oral treatments for acute pain in patients with herpes zoster, although there have been recommendations for pain control according to the degree of pain [18].

For mild to moderate pain, we recommended consideration of paracetamol, non-steroidal anti-inflammatory drugs (NSAIDs), or mild opioids such as tramadol. If pain is moderate to severe, opioids such as oxycodone and morphine can be given [11]. There is no role for adjuvant pain therapy with oral glucocorticoids [18] or tricyclic antidepressants [19].

Evidence on the usefulness of gabapentin for pain in acute herpes neuralgia remains conflicting [20,21,22,23].

#### 3.5.3. Post-Herpetic Neuralgia

Post-herpetic neuralgia can be treated with topical or systemic agents. Topical agents such as topical lidocaine 2% gel 4 times/day [24,25] and topical capsaicin 0.05% ointment 4 times/day [26,27] can be utilized.

Systemic agents as such opioid analgesics [25], tricyclic antidepressants [28,29], and anticonvulsants [30,31,32] can also be used. Common options include tramadol for opioids, amitriptyline or nortriptyline for tricyclic antidepressants, and gabapentin or pregabalin for anticonvulsants. The use of opioids for pain should be balanced against the risk of tolerance, dependence, and potential for abuse. The summary of treatment options in post-herpetic neuralgia can be found in Table 5.

### 3.6. Prevention of Post-Herpetic Neuralgia

There is currently limited literature on effective strategies for preventing PHN. A recent systemic review and network meta-analysis [33] have shown that the incidence of PHN was decreased with the following therapies, in decreasing order of effectiveness:

1. Epidural block with local anesthetics and steroids (EPI-LSE);
2. Antiviral agents with subcutaneous injection of local anesthetics and steroids (AV + sLS);
3. Antiviral agents with intracutaneous injection of local anesthetics and steroids (AV + iLS);
4. Antiviral agents with anti-epileptics and stellate ganglion block using local anesthetics and steroids;
5. Antiviral agents with anti-epileptics and paravertebral block using local anesthetics and steroids.

The above measures have shown effectiveness in reducing the incidence of PHN at 1, 3, and 6 months but additional studies are needed to look at the longer-term efficacy of these measures.

### 3.7. Vaccination Strategies

Herpes zoster is a disease that can be prevented with a vaccine. The Society of Infectious Disease in Singapore [34] advises vaccination for adults over 50 years old, as well as for adults over 19 years old who are at higher risk due to immunodeficiency or immunosuppression.

There is no upper age limit for receiving the shingles vaccine. Patients can obtain the vaccine any time after having shingles but it is generally recommended to wait until the shingles rash has disappeared before getting vaccinated. There are currently two types of zoster vaccines (Table 6):

1. A live attenuated vaccine (designated zoster vaccine line [ZVL], sold as Zostavax). It is a one-dose live attenuated vaccine that boosts VZV-specific cell-mediated immunity;
2. Inactivated adjuvant recombinant zoster vaccine (RZV), sold as Shringrix, was approved in 2021 and is administered in a series of 2 doses at 2 to 6 months apart. RZV provides 97.2% overall vaccine efficacy and 91.2% protection against postherpetic neuralgia in immunocompetent adults aged ≥50 years old [35]. There is currently no recommendation for booster with some evidence that the clinical benefit of the vaccine in adults ≥50 is sustained up to 10 years after vaccination [35].

**Table 6:** Herpes zoster vaccination.

	Live Attenuated (Zostavax®)	Recombinant, Adjuvanted (Shingrix®)
Description	Each dose contains at least 19,400 plaque-forming units of the attenuated VZV	Each dose contains a lyophilized varicella zoster virus glycoprotein E (gE) antigen component, to be reconstituted with the accompanying vial of AS01B adjuvant suspension component
Summary of evidence	Clinical trials involving elderly individuals who had not previously experienced zoster showed a 50% to 70% decrease in the occurrence of zoster [36], along with the following reductions: 61.1% reduction in the burden of illness from herpes zoster [37] 66.5% reduction in the incidence of postherpetic neuralgia (PHN) [37] 51.3% reduction in the incidence of herpes zoster [37]	A randomized placebo-controlled study involving older adults (aged 50 and above) found that the vaccine was 97.2% [37] effective overall in preventing zoster and reduced the incidence of postherpetic neuralgia (PHN) by 91.2% [35]
Indication	Prevention of zoster in adults aged 50 years and older	Prevention of zoster in adults aged 50 years and older
Schedule	Single dose	Administer 2 doses (0.5 mL each) 2–6 months apart
Administration	Subcutaneous injection	Intramuscular injection
Common adverse events	Redness, pain, and swelling at the injection site Fever, headache, body aches, malaise, nausea, and itching	Injection site pain, redness, and swelling Myalgia, fatigue, headache, shivering, fever, and gastrointestinal symptoms
Contraindications	Anaphylaxis to any vaccine component or a previous dose, including neomycin and gelatin Immunocompromised state, except those with leukemia in remission and those who have not received chemotherapy or radiation for at least 3 months [38]	Anaphylaxis to any component of the vaccine or a previous dose
Precautions	Vaccination should be delayed for individuals experiencing a severe acute illness There is a minor risk that the vaccine virus could be transmitted from vaccinated individuals to those who are susceptible	Before administering the vaccine, check the patient’s immunization history for any potential vaccine sensitivities and previous adverse reactions to vaccinations. Ensure that suitable medical treatment and supervision are available to handle any possible anaphylactic reactions
Pregnancy and breastfeeding	Category C in pregnancy Pregnant women should not be vaccinated and pregnancy should be avoided for three months after receiving the vaccine Breastfeeding mothers should be cautious when receiving the vaccine, as the varicella-zoster virus (VZV) may be present in breast milk	There are no available human data to establish whether there is vaccine-associated risk in pregnant women. It is not known whether the vaccine is excreted in human milk

## 4. Conclusions

The diagnosis of herpes zoster is clinical and can be aided with laboratory investigations. Antivirals should be started early, preferably within 72 h of the onset of herpes zoster to reduce the severity and duration of the condition and decrease the intensity of pain. In patients with a high risk of post-herpetic neuralgia, early initiation of anticonvulsants or tricyclic antidepressants can be considered.

The RZV is effective in reducing the incidence of herpes zoster and should be considered in patients who are at risk of herpes zoster. Procedures such as epidural blocks and subcutaneous or intracutaneous injections of local anesthetics and steroids can be considered for patients with a high risk of PHN to reduce its incidence.

## Figures and Tables

**Table 1:** Risk factors for the development of herpes zoster.

Psychological stress Advancing age Immunosuppression Human immunodeficiency virus (HIV) / acquired immunodeficiency syndrome (AIDS) Malignancies Post-transplant Chronic diseases Autoimmune diseases (systemic lupus erythematosus, rheumatoid arthritis, and inflammatory bowel diseases) Chronic respiratory diseases, e.g., asthma and chronic obstructive pulmonary disease Chronic renal disease Diabetes Physical trauma Local trauma may be associated with herpes zoster—reactivation of latent virus possibly via stimulation of local sensory nerves

**Table 2:** Risk factors for post-herpetic neuralgia.

Older age > 50 years old Greater pain severity with acute herpes zoster Greater rash severity Ophthalmic localization of the acute herpes zoster rash Presence of a prodrome

**Table 3:** Selected complications of herpes zoster infection.

Complications	Manifestations
Post herpetic neuralgia	Burning pain persisting > 3 months after resolution of rash
Herpes zoster ophthalmicus	‘Hutchinson’s sign’ or vesicular lesions over tip of the nose Involvement of the ophthalmic division of the V nerve
Ramsay Hunt syndrome	Unilateral painful vesicles on the external auditory canal, otalgia, and facial paralysis Involvement of geniculate ganglion
Others	Acute retinal necrosis—may present with acute onset of vision loss, redness, photophobia, pain, floaters, and flashes. Classically, there is posterior segment involvement including vitreous inflammation, retinal vascular arteriolitis, and peripheral retinitis Keratitis and uveitis Aseptic meningitis, encephalitis, and transverse myelitis Urinary retention and hematuria due to bladder wall involvement Diaphragmatic involvement due to phrenic nerve palsy

**Table 4:** Antiviral medications for herpes zoster infection.

Medication	Oral Dosage *	Duration ^#^
Acyclovir	800 mg 5 times daily	7 days
Valacyclovir	1000 mg 3 times daily	7 days
Famciclovir	500 mg 3 times daily	7 days

* Required dose adjustment in patients with renal insufficiency. ^#^ Duration extended for immunocompromised hosts based on clinical assessment.

**Table 5:** Summary of treatment options in post-herpetic neuralgia.

Treatment	Recommendations	Precautions
Topical Therapies		
Topical lidocaine	Consider topical treatment if pain is mild or when systemic agents are contraindicated, e.g., in elderly patients.	Local irritation might be seen
Topical capsaicin	Consider topical treatment if pain is mild or when systemic agents are contraindicated, e.g., in elderly patients.	Local irritation might be seen
Oral Therapies		
Anticonvulsants (1) Gabapentin (2) Pregabalin	Consider for use in patients with moderate to severe PHN.	Side effects of giddiness, somnolence, and peripheral oedema may be seen
Tricyclic antidepressants (1) Amitriptyline (2) Nortriptyline	Consider for use in patients with moderate to severe PHN.	Side effects of sedation, cognitive impairment, dry mouth, and giddiness may be seen. Contraindicated in patients with heart disease, glaucoma, urinary retention, and high risk of suicide death.
Opioid analgesics	May be considered in patients with contraindications to tricyclic antidepressants or anticonvulsants.	Might lead to increased dependence and addiction. Use with caution in patients with renal impairment

## Data Availability

The data that support the findings of this study are available from the corresponding author upon reasonable request.
